# Hypotension during propofol sedation for colonoscopy: a retrospective exploratory analysis and meta-analysis

**DOI:** 10.1016/j.bja.2021.10.044

**Published:** 2021-12-13

**Authors:** J. Robert Sneyd, Anthony R. Absalom, Clemens R.M. Barends, Jordan B. Jones

**Affiliations:** 1 Faculty of Medicine and Dentistry, University of Plymouth, Plymouth, UK; 2 Department of Anesthesiology, University Medical Center Groningen, University of Groningen, Groningen, the Netherlands; 3 College of Osteopathic Medicine, Rocky Vista University, Ivins, UT, USA

**Keywords:** colonoscopy, endoscopy, hypotension, midazolam, propofol, sedation

## Abstract

**Background:**

Intraoperative and postoperative hypotension occur commonly and are associated with organ injury and poor outcomes. Changes in arterial blood pressure (BP) during procedural sedation are not well described.

**Methods:**

Individual patient data from five trials of propofol sedation for colonoscopy and a clinical database were pooled and explored with logistic and linear regression. A literature search and focused meta-analysis compared the incidence of hypotension with propofol and alternative forms of procedural sedation. Hypotensive episodes were characterised by the original authors' definitions (typically systolic BP <90 mm Hg).

**Results:**

In pooled individual patient data (*n*=939), 36% of procedures were associated with episodes of hypotension. Longer periods of propofol sedation and larger propofol doses were associated with longer-lasting and more-profound hypotension. Amongst 380 patients for whom individual BP measurements were available, 107 (28%) experienced systolic BP <90 mm Hg for >5 min, and in 89 (23%) the episodes exceeded 10 min. Meta-analysis of 18 RCTs identified an increased risk ratio for the development of hypotension in procedures where propofol was used compared with the use of etomidate (two studies; *n*=260; risk ratio [RR] 2.0 [95% confidence interval: 1.37–2.92]; *P*=0.0003), remimazolam (one study; *n*=384; RR 2.15 [1.61–2.87]; *P*=0.0001), midazolam (14 studies; *n*=2218; RR 1.46 [1.18–1.79]; *P*=0.0004), or all benzodiazepines (15 studies; *n*=2602; 1.67 [1.41–1.98]; *P*<0.00001). Hypotension was less likely with propofol than with dexmedetomidine (one study; *n*=60; RR 0.24 [0.09–0.62]; *P*=0.003).

**Conclusions:**

Hypotension is common during propofol sedation for colonoscopy and of a magnitude and duration associated with harm in surgical patients.

**Editor's key points**

• Hypotension during procedural sedation is not well characterised. The authors aggregated individual patient data from five RCTs and a clinical database to explore this issue.
• More than a third of subjects receiving propofol sedation suffered hypotension, with duration and magnitude comparable to those associated with harm during surgery.
• Future research should attempt to confirm these findings with other data sources and to explore the relationship, if any, between hypotension and adverse outcomes.

Recently, intraoperative and postoperative hypotension have been identified as major correlates of adverse outcomes and a target for research and interventions. Gregory and colleagues^1^ commented, ‘…we believe hypotension in the operating room is a serious public health issue, and should not be ignored in any age group. We suggest there is an urgent and currently unmet need for prospective interventional studies focused on its prevention’. Brief (>5 min) decreases from baseline of systolic BP by 41–50 mm Hg correlate with treble the odds of myocardial infarction.^2^ Five minutes with systolic BP <90 mm Hg has been suggested as a threshold for associated myocardial and renal injury.^3^ Ten minutes or more with MAPs below 80 mm Hg is associated with increased mortality, and the risk increases with longer exposures and lower pressures.^4^

BP changes during and after procedural sedation have been less well documented, and their significance, if any, has not been determined. Propofol is widely used for procedural sedation by anaesthesiologists and increasingly by non-anaesthesiologists. Rapid onset, ease of titration, and swift clear-headed recovery provide an acceptable patient experience and operator satisfaction, and supporting efficient patient throughput. Propofol use underpins increased participation by anaesthesia services in outpatient colonoscopy (from 16% to 58% in the USA during the decade 2006–15).^5^ However, propofol decreases systemic vascular resistance and is associated with hypotension during and after induction of anaesthesia.^6^^,^^7^

Colonoscopy is a high-volume investigation^8^ usually performed under procedural sedation. In a Melbourne series of 2132 patients having endoscopy (1767 elective; 365 emergencies; median age 60 yr; 42% ASA physical status 3–5), 98% received propofol.^9^ Significant unplanned events occurred in 23%, including ‘significant hypotension’ (systolic BP <90 mm Hg and requiring i.v. fluid bolus or vasopressor) in 11% of elective cases and 17% of emergencies. The 1268 (60%) patients who had colonoscopy were twice as likely to experience significant unplanned intraoperative events than patients who did not have colonoscopy. Patients having colonoscopy have patient- and procedure-related factors (such as administration of bowel preparation,^10^ age, and comorbidities) that may predispose them to hypotension and are therefore a rational group to observe for sedation-induced hypotension.

We hypothesised that the propensity of propofol to promote hypotension may be expressed during procedural sedation. We used pooled data to identify the prevalence and magnitude of hypotension during propofol sedation for colonoscopy (primary goal). Using a literature search and a focused meta-analysis, we investigated whether episodes of hypotension are more frequent with propofol than with alternative forms of sedation for colonoscopy (secondary goal).

## Methods

### Retrospective analysis of hypotension in published studies

We performed a reanalysis of anonymised individual patient data from six published studies of propofol sedation for colonoscopy, including five RCTs^11^, ^12^, ^13^, ^14^, ^15^ and a retrospective cohort study.^16^ Data from the five trials were shared under a data transfer agreement between Melbourne Health and the University of Plymouth. A sixth data set from Dutch patients receiving propofol sedation for colonoscopy within a hospital sedation service^16^ was accessed locally. The data were acquired during a service evaluation approved by the Institutional Review Board (METc-number 2018/106). UK NHS and University Research Ethics services both advised that they did not need to approve this reanalysis of anonymous non-UK data.

From the study data, we extracted the patient's sex, age, weight, ASA physical status, duration of sedation, and total dose of propofol administered. Patients were characterised as experiencing hypotension (or not) by the original investigators.^11^, ^12^, ^13^, ^14^, ^15^, ^16^ Hypotension was defined as a lowest measured systolic BP below 90 mm Hg, a diastolic BP below 50 mm Hg, or a 20% decrease of systolic BP from baseline.

Where individual values of systolic BP were available, we quantified the degree of hypotension by determining the area under the curve (AUC) below 90 mm Hg. AUC (mm Hg × min) was calculated as the sum of the products of the duration spent below the 90 mm Hg threshold and the difference between these measurements and the threshold. The time-weighted average of the hypotensive episodes was calculated as AUC divided by the duration of sedation.

In our second analysis, we used regression models to test possible associations of the candidate variables sex; age; weight; ASA physical status; procedure/sedation duration; and total dose of propofol with the (i) occurrence of hypotension, (ii) lowest systolic BP, and (iii) duration of systolic BP <90 mm Hg. Assumption tests for linearity and multicollinearity were performed for each of the three models. *P*-values <0.05 were considered significant. All analyses were performed with IBM SPSS Statistics, version 23.0.0.3 (IBM, Armonk, NY, USA).

### Three regression analyses were performed

Analysis 1 was a binary logistic regression analysis. In this analysis, we investigated the occurrence of one or more episodes of hypotension (defined by the original investigators) and its relationship with the following clinically relevant covariates: total dose of propofol given during the procedure (in mg kg^−1^), duration of the sedation, age, sex, and ASA physical status.

Analysis 2 was a linear regression analysis investigating the relationship between the lowest systolic BP measured during the procedure and the following clinically relevant covariates: total dose of propofol given during the procedure (in mg kg^−1^), duration of the sedation, age, sex, and ASA physical status.

Analysis 3 investigated the relationship of the five clinically relevant covariates (total dose of propofol given during the procedure [in mg kg^−1^], duration of the sedation, age, sex, and ASA physical status) with the total time spent with systolic BP below 90 mm Hg during the procedure.

### Meta-analysis: literature search, data extraction, and analysis

We searched PubMed, Embase, and Web of Science in July 2021 to identify publications reporting RCTs, in which adults having elective colonoscopy were randomised to propofol or non-propofol sedation. We extended our search though publications cited in these reports and relevant narrative and systematic reviews, searches for full papers by authors who had published abstracts, and other *ad hoc* searching. Self-administered (patient controlled) sedation and trials, which did not report hypotensive episodes as an outcome, were excluded. Our search strategy is described in the Supplementary material. Two authors (JBJ and JRS) at different institutions searched independently with local librarian support. After screening titles and abstracts, electronic reprints of likely publications were shared and reviewed by both authors. We attempted to contact the authors of papers reporting apparently suitable trials that did not report hypotension. Foreign language publications were assessed using Google Translate™, translate.google.co.uk, or fluent speakers of the relevant language. Data, including patient characteristics, drugs, and doses and hypotensive episodes, were extracted into a spreadsheet by JBJ and validated by JRS. Our search and meta-analysis were limited to establishing the proportion of patients experiencing hypotensive episodes and their association with a randomly allocated sedation scheme.

Validated data were transferred to specialist software (Review Manager 5.4; www.cochrane.org) for statistical analysis and preparation of forest plots. Risk-of-bias assessment was undertaken using the Cochrane Risk of Bias 2 tool for RCTs. The RoB 2 scores were used to separate the studies into three categories: ‘low risk’, ‘some concerns’, and ‘high risk’.

Treatment arms were categorised by hypnotic, regardless of opioid use. Propofol sedation was compared with etomidate or dexmedetomidine sedation, with all benzodiazepines, and with midazolam and remimazolam. Risk ratios (RRs) and 95% confidence intervals were calculated using the Mantel–Haenszel fixed-effect method of meta-analysis.

## Results

### Retrospective analysis of individual patient data

The combined data set described 985 patient episodes of elective colonoscopy with propofol sedation^11^, ^12^, ^13^, ^14^, ^15^, ^16^; 46 episodes were excluded because of missing or artifactual data, leaving 939 available for analysis (Table 1). Constraints of anonymisation mean that our data may include a small number of patients who had more than one procedure, and we have treated each episode as a separate patient. Patient characteristics are summarised in Table 2. These patients were aged 49 (16) yr and weighed 77 (17) kg; mean (standard deviation [sd]). For 340 patients,^14^, ^16^ the duration of sedation was not available, so we substituted duration of procedure. Calculated on this basis, the mean (sd) duration of sedation was 32 (21) min and the mean (sd) propofol infusion rate was 170 (150) mcg kg^−1^ min^−1^. For 599 procedures, where both procedure duration and sedation duration were recorded,^11^, ^12^, ^13^, ^15^ the difference was 4.0 (3.6) min; mean (sd). In an alternate analysis, we added 4.0 min to the procedure time for these 340 patients, and the results were similar (Supplementary material).

**Table 1:** Five RCTs and one observational study with data characterising arterial BP during propofol sedation for colonoscopy. BIS, *bispectral index* SBP, systolic BP; TCI, target-controlled infusion.

Study	Study design and intervention	Number of procedures available for analysis	Available BP data	Measurement interval (min)	BP information used in analysis	Hypotension criteria used in analysis
Padmanabhan and colleagues^11^ (2009)	RCT; propofol sedation with or without midazolam with or without fentanyl	200	Lowest SBP during colonoscopy	2.5	Lowest SBP during colonoscopy	Hypotension=lowest SBP <90 mm Hg
Allen and colleagues^12^ (2015)	RCT; light *vs* deep (BIS guided) sedation with propofol and fentanyl	199	Occurrence of hypotension	Unknown	Hypotension yes/no	Hypotension=MAP 20% below baseline, or systolic pressure <90 mm Hg, or diastolic pressure <50 mm Hg
Leslie and colleagues^13^ (2006)	RCT; propofol sedation and 1.5 or 15 ml kg^−1^ of Hartmann's solution	160	Lowest SBP during colonoscopy	2.5	Lowest SBP; hypotension yes/no	Hypotension=lowest SBP below 90 mm Hg
Leslie and colleagues^14^ (2016)	RCT; propofol sedation and 2 or 20 ml kg^−1^ of Plasma-Lyte 148	150	Lowest SBP during colonoscopy	2.5	Lowest SBP; hypotension yes/no	Hypotension=lowest SBP below 90 mm Hg
Stonell and colleagues^15^ (2006)	RCT; patient-controlled *vs* anaesthetist-controlled propofol sedation	40	All BP measurements during colonoscopy	3	Lowest SBP; hypotension yes/no	Hypotension=lowest SBP below 90 mm Hg
Barends and colleagues^16^ (2020)	Observational study of TCI sedation with propofol and remifentanil	2937 (various procedures; 190 colonoscopies)	All BP measurements during colonoscopy	2.5	Lowest SBP; hypotension yes/no	Hypotension=lowest SBP below 90 mm Hg

**Table 2:** Patient characteristics. Data are mean (standard deviation) [range] or *n* (%), as appropriate.

	*N*	Age	Weight	ASA 1, *n* (%)	ASA 2, *n* (%)	ASA 3, *n* (%)	Male, *n* (%)	Female, *n* (%)
Padmanabhan and colleagues^11^ (2009)	200	50 (15) [19–82]	78 (19) [45–170]	72 (36)	98 (49)	30 (15)	93 (47)	107 (54)
Allen and colleagues^12^ (2015)	199	51 (15) [20–84]	80 (18) [45–150]	71 (36)	90 (45)	38 (19)	103 (52)	96 (48)
Leslie and colleagues^13^ (2006)	160	51 (15) [21–83]	77 (16) [41–130]	57 (36)	87 (54)	16 (10)	87 (54)	73 (46)
Leslie and colleagues^14^ (2016)	150	50 (16) [19–89]	77 (16) [37–125]	37 (25)	86 (57)	27 (18)	84 (56)	66 (44)
Stonell and colleagues^15^ (2006)	40	46 (13) [21–79]	81 (22) [52–183]	19 (48)	20 (50)	1 (3)	32 (80)	8 (20)
Barends and colleagues^16^ (2020)	190	46 (18) [18–83]	72 (15) [45–141]	16 (8)	143 (75)	31 (16)	46 (24)	144 (76)
Total	939			272 (29)	524 (56)	143 (15)	445 (47)	494 (53)

BP data varied between studies from raw measurements at 2.5 or 3 min intervals to binary indications that hypotension had occurred (Table 1). Seven hundred and forty procedures could be analysed based on the lowest recorded systolic BP.

**Fig 1:**
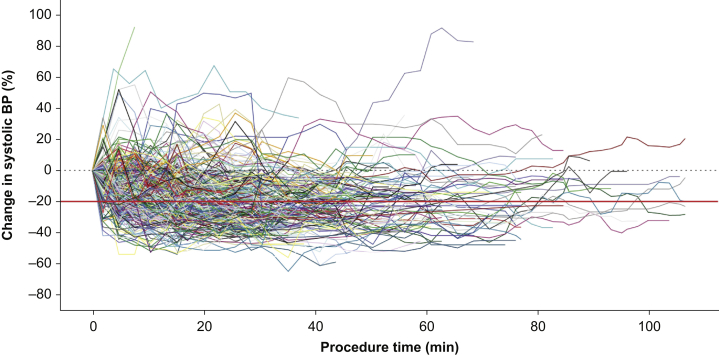
Individual percentage change from baseline (black dotted line) in systolic BP for 380 patients receiving propofol sedation for elective colonoscopy.^14^, ^15^, ^16^ Reference line (red solid line) indicates 20% reduction in BP.

Changes in BP were common during sedation, with many patients experiencing extended periods of hypotension (Fig. 1). One or more episodes of hypotension occurred during 333/939 (35%) procedures.

The individual BP measurements of 380 patients were included in an analysis of the duration and extent of hypotension. One hundred and seventy-six (46%) experienced one or more episodes of hypotension. The lowest recorded SBP was 97 (17) mm Hg for all 380 patients and 80 (8) mm Hg for the 176 with a recorded episode of hypotension; mean (sd). One hundred and seven (28%) experienced a total of 158 hypotensive episodes lasting longer than 5 min, and in 89 (23%) a total of 101 hypotensive episodes exceeded 10 min. In patients experiencing hypotension, the duration was 13 [5–23] min, AUC 68 [3–836] mm Hg min^−1^, and time-weighted average 2.0 [0.04–20] mm Hg; data are median [range].

Three regression analyses were performed. The different characteristics of available BP data (Table 1) meant that some studies were excluded from Analysis 2 and Analysis 3 (Table 3).

**Table 3:** Inclusion of patients into regression analysis. SBP, systolic BP.

	Total patients	Number of procedures used in regression analysis 1 (occurrence of hypotension)	Number of procedures used in regression analysis 2 (lowest SBP measured)	Number of procedures used in regression analysis 3 (minutes below SBP=90)
Padmanabhan and colleagues^11^ (2009)	200	200	200	Not available
Allen and colleagues^12^ (2015)	199	199	Not available	Not available
Leslie and colleagues^13^ (2006)	160	160	160	Not available
Leslie and colleagues^14^ (2016)	150	150	150	150
Stonell and colleagues^15^ (2006)	40	40	40	40
Barends and colleagues^16^ (2020)	190	190	190	190
Total included in analysis	939	939	740	380

Analysis 1, exploring the relationship between the occurrence of hypotension and clinically relevant covariates by binary logistic regression, included data from 939 patients (Table 4). We found a positive relationship between the total dose of propofol given and the odds of developing an episode of hypotension; *P*=0.023. Likewise, the duration of propofol administration was positively related to the odds of developing hypotension; *P*≤0.001.

**Table 4:** Analysis 1, binary logistic regression exploring the occurrence of one or more episodes of hypotension in relation to clinically relevant covariates: total dose of propofol, duration of propofol administration, age, sex, and ASA physical status. In this analysis, all 939 patients were included. CI, confidence interval.

	Odds ratio	95% CI		*P*-value
		Lower bound	Upper bound	
Total dose of propofol (mg kg^−1^)	1.079	1.01	1.152	0.023
Duration of propofol administration (min)	1.021	1.012	1.030	<0.001
Age (yr)	0.998	0.988	1.008	0.665
Male sex	0.94	0.709	1.247	0.668
ASA physical status 1				0.591
ASA physical status 2	1.045	0.744	1.468	0.799
ASA physical status 3	1.269	0.784	2.053	0.332

Analysis 2, exploring the relationship between the lowest systolic BP measured during the procedure and clinically relevant covariates by linear regression, used data from 740 patients (Table 5). We found that the total dose of propofol (*P*=0.007), the duration of the procedure (*P*<0.001), and the age of the patient (*P*<0.001) were significantly associated with the lowest measured systolic BP. The correlation between age and lowest measured systolic BP was positive (i.e. the minima were higher in the older patients). We explored this with some additional regressions (Supplementary material), which demonstrate that the older patients had higher baseline pressures, were given smaller propofol doses, but experienced a greater drop in pressure.

**Table 5:** Analysis 2, linear regression analysis exploring the relationship between the lowest systolic BP measured during the procedure and clinically relevant covariates: total dose of propofol, duration of propofol administration, age, sex, and ASA physical status. In this analysis, 740 patients were included. CI, confidence interval; se, standard error.

	Coefficients	se	95% CI		*P*-value
			Lower bound	Upper bound	
Total dose of propofol (mg kg^−1^)	–0.731	0.269	–1.259	–0.202	0.007
Duration of propofol administration (min)	–0.212	0.033	–0.277	–0.147	<0.001
Age (yr)	0.153	0.042	0.071	0.235	<0.001
Male sex	1.217	1.201	–1.142	3.575	0.312
ASA physical status	–0.737	1.030	–2.759	1.285	0.474

Analysis 3, exploring the relationship between the total time spent with systolic BP below 90 mm Hg during the procedure and clinically relevant covariates by linear regression, used data from 380 patients (Table 6). We found that the total dose of propofol (*P*=0.008) and the duration of the procedure (*P*<0.001) were significantly associated with the total time spent with systolic BP below 90 mm Hg during the procedure.

**Table 6:** Analysis 3, linear regression analysis exploring the relationship between the total time spent with a systolic BP below 90 mm Hg during the procedure and clinically relevant covariates: total dose of propofol, duration of propofol administration, age, sex, and ASA physical status. In this analysis, 380 patients were included. CI, confidence interval; se, standard error.

	Coefficients	se	95% CI		*P*-value
			Lower bound	Upper bound	
Total dose of propofol (mg kg^−1^)	0.572	0.213	0.153	0.992	0.008
Duration of propofol administration (min)	0.225	0.025	0.175	0.275	<0.001
Age (yr)	–0.012	0.036	–0.083	0.059	0.743
Male sex	0.307	1.126	–1.907	2.521	0.785
ASA physical status	0.613	0.996	–1.345	2.571	0.538

### Meta-analysis

**Fig 2:**
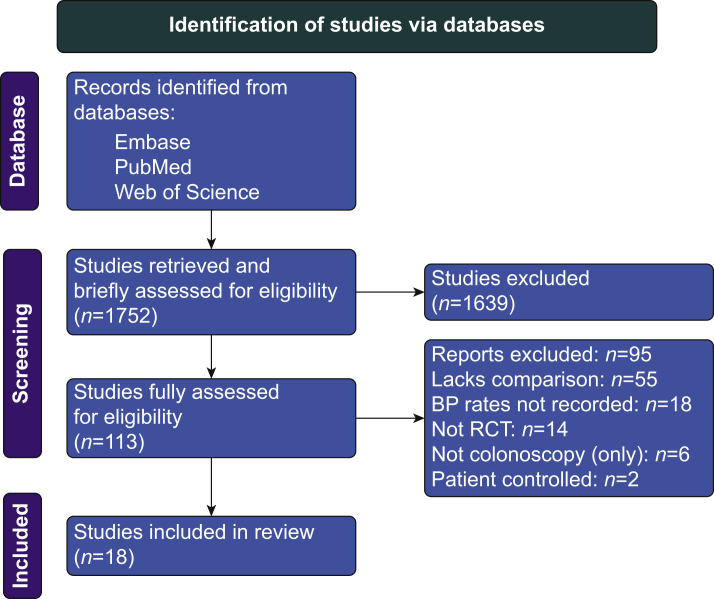
Search process shown as a Preferred Reporting Items for Systematic Reviews and Meta-Analyses (PRISMA) flow chart.

Our literature search yielded 18 eligible trials (Fig. 2). Propofol was compared with midazolam in 14 trials, remimazolam in one trial, etomidate in two trials, and dexmedetomidine in one trial. Patient characteristics in included studies are summarised in Table 7. Drug doses and hypotension are in Table 8. Risk of Bias 2 grades are in the Supplementary material.

**Table 7:** Patient characteristics in included studies. Data are mean (standard deviation) or median [range]. Minor inconsistencies in data formats reflect their presentation in the publications from which they were extracted. N/A, not available.

Author (publication year)	Sedation arms	Group size, *N*	ASA grade (%)	Age (yr)	Female (%)	Weight (kg)	BMI (kg m^−2^)
Adigun and colleagues^17^ (2019)	Propofol and fentanyl	31	1: 36	61 (11)	42	69 (14)	N/A
			2: 65				
			3: 0				
			4: 0				
	Midazolam and pentazocine	31	1: 39	62 (13)	48	75 (17)	N/A
			2: 58				
			3: 3				
			4: 0				
Bastaki and colleagues^18^ (2013)	Propofol	50	1: 40	59 (11)	56	74 (13)	27 (5)
			2: 60				
			3/4: 0				
	Midazolam and fentanyl	50	1: 38	58 (12)	46	78 (16)	26 (4)
			2: 62				
			3/4: 0				
Chen and colleagues^19^ (2020)	Propofol and fentanyl	190	1: 76	44 (11)	54	64 (11)	23 (3)
			2: 23				
			3: 0				
			4: 0				
	Remimazolam and fentanyl	194	1: 81	45 (12)	62	63 (11)	23 (3)
			2: 19				
			3: 0				
			4: 0				
Eberl and colleagues^20^ (2014)	Propofol and alfentanil	60	1: 32	N/A	58	79 (16)	27 (6)
			2: 48				
			3: 20				
			4: 0				
	Midazolam and fentanyl	60	1: 27	N/A	50	77 (16)	26 (5)
			2: 58				
			3: 15				
			4: 0				
Ekmekçi and colleagues^21^ (2017)	Propofol and remifentanil	50	N/A	54 (10)	54	73 (16)	N/A
	Midazolam and meperidine	50	N/A	57 (14)	62	72 (13)	N/A
Fanti and colleagues^22^ (2015)	Propofol and fentanyl	35	1: 54	57 (14)	37	N/A	25 (6)
			2: 46				
			3/4: 0				
	Midazolam and fentanyl	35	1: 57	59 (12)	43	N/A	23 (5)
			2: 43				
			3/4: 0				
Gurbulak and colleagues^23^ (2014)	Propofol, midazolam, and fentanyl	62	1: 72	46 [19–78]	59	N/A	28 (5)
			2: 23				
			3: 5				
			4: 0				
	Midazolam, meperidine, and fentanyl	62	1: 54	48 [19–88]	55	N/A	27 (6)
			2: 41 3: 5				
			4: 0				
Heuss^24^ (2012)	Propofol and alfentanil	41	1: 32	62 (13)	51	N/A	N/A
			2: 37				
			3: 32				
			4: 0				
	Midazolam and alfentanil	42	1: 29	62 (13)	55	N/A	N/A
			2: 38				
			3: 33				
			4: 0				
Karanth and colleagues^25^ (2018)	Propofol and fentanyl	30	N/A	46 (13)	40	56 (8)	N/A
	Dexmedetomidine and fentanyl	30	N/A	47 (12)	27	58 (7)	N/A
Kim and colleagues^26^ (2021)	Propofol	89	1: 60	61 (9)	53	N/A	24 (3)
			2: 40				
			3: 0				
			4: 0				
	Midazolam (bolus) and meperidine	89	1: 56	58 (14)	43	N/A	24 (4)
			2: 44				
			3: 0				
			4: 0				
	Midazolam (titrated) and meperidine	89	1: 53	59 (12)	48	N/A	24 (3)
			2: 47				
			3: 0				
			4: 0				
Lee and colleagues^27^ (2019)	Propofol and midazolam	100	1: 43	57 (15)	50	N/A	24 (4)
			2: 51				
			3: 6				
			4: 0				
	Etomidate and midazolam	100	1: 45	58 (16)	46	N/A	23 (3)
			2: 48				
			3: 7				
			4: 0				
Padmanabhan and colleagues^28^ (2017)	Propofol	300	1: 29	61 (10)	46	N/A	30 (6)
			2: 56				
			3: 15				
			4: 0				
	Midazolam and fentanyl	300	1: 29	61 (9)	49	N/A	30 (6)
			2: 55				
			3: 16				
Paspatis and colleagues^29^ (2002)	Propofol and midazolam	64	1/2: 81	61 (11)	48	N/A	N/A
			>3: 19				
	Midazolam and meperidine	56	1/2: 82	60 (12)	48	N/A	N/A
			>3: 18				
Schroeder and colleagues^30^ (2016)	Propofol	126	N/A	58 (13)	48	N/A	N/A
	Midazolam and fentanyl	136	N/A	58 (14)	44	N/A	N/A
Sipe and colleagues^31^ (2002)	Propofol	40	1.3 (0.4)	52 (11)	48	83 (22)	N/A
	Midazolam and meperidine	40	1.3 (0.5)	54 (14)	53	82 (18)	N/A
Steenholdt and colleagues^32^ (2020)	Propofol	63	1.4 (0.5)	42 (13)	41	N/A	25 (4)
	Midazolam and fentanyl	67	1.4 (0.5)	41 (14)	57	N/A	24 (4)
Toklu and colleagues^33^ (2009)	Propofol and remifentanil	30	1: 10	51 (11)	57	68 (11)	N/A
			2: 20				
			3: 0				
			4: 0				
	Etomidate and remifentanil	30	1: 13	48 (11)	60	72 (12)	N/A
			2: 17				
			3: 0				
			4: 0				
Ulmer and colleagues^34^ (2003)	Propofol	50	1.4 (0.5)	56 (11)	42	83 (15)	N/A
	Midazolam and fentanyl	50	1.3 (0.6)	55 (12)	50	82 (21)	N/A

**Table 8:** Drug doses and hypotension in included studies. Data are mean (standard deviation) or median [range].

Author (publication year)	Propofol dose (mg)	Comparator	Comparator dose (mg)	Opioid	Opioid dose (mcg)	Hypotension, *n* (%)
Adigun and colleagues^17^ (2019)	471 (10)			Fentanyl	57 (13)	6 (19)
		Midazolam	2.5	Pentazocine	17.65 (5.8)	2 (7)
Bastaki and colleagues^18^ (2013)	153 (53)					3 (6)
		Midazolam	7.6 (2.7)	Fentanyl	50	0 (0)
Chen and colleagues^19^ (2020)	96			Fentanyl	64 (11)	97 (51)
		Remimazolam	5	Fentanyl	63 (11)	46 (24)
Eberl and colleagues^20^ (2014)	442 (177)			Alfentanil	232 (127)	56 (93)
		Midazolam	3.9 (1.5)	Fentanyl	67 (29)	21 (35)
Ekmekçi and colleagues^21^ (2017)	100 mcg kg^−1^ min^−1^			Remifentanil	73.1	2 (4)
		Midazolam	2	Meperidine	20 000	1 (2)
Fanti and colleagues^22^ (2015)	110 (47)			Fentanyl	71 (15)	1 (1)
		Midazolam	2.9 (1.0)	Fentanyl	72 (19)	3 (4)
Gurbulak and colleagues^23^ (2014)	118 (32)	Midazolam	2.5	Fentanyl	50	22 (36)
		Midazolam	6.5 (1.1)	Fentanyl/meperidine	50/30 500 (5600)	17 (27)
Heuss^24^ (2012)	131 [70–260]			Alfentanil	4 mcg kg^−1^	17 (42)
		Midazolam	5 [4–7]	Alfentanil	4 mcg kg^−1^	17 (41)
Karanth and colleagues^25^ (2018)	[2–3] mg kg^−1^+infusion			Fentanyl	29	4 (13)
		Dexmedetomidine	1 mcg kg^−1^+infusion	Fentanyl	29	17 (57)
Kim and colleagues^26^ (2021)	82 (30)					1 (1)
		Midazolam (bolus)	4.8 (1.5)	Meperidine	50 000	0 (0)
		Midazolam (titrated)	4.4 (1.5)	Meperidine	50 000	1 (1)
Lee and colleagues^27^ (2019)	0.5 mg kg^−1^	Midazolam	2.9 (0.7)			42 (42)
		Etomidate/midazolam	0.1 mg kg^−1^/3.0 (0.6)			27 (27)
Padmanabhan and colleagues^28^^,^^35^ (2017; 2020)	251.3 (76.9)					3 (1)
		Midazolam	6.9 (2.1)	Fentanyl	149 (64)	8 (3)
Paspatis and colleagues^29^ (2002)	80 [40–150]	Midazolam	[2–3]			24 (38)
		Midazolam	5 [3–7]	Meperidine	75 000 [50 000–125 000]	17 (30)
Schroeder and colleagues^30^ (2016)	341 (122.8)					3 (2)
		Midazolam	5.7 (1.4)	Fentanyl	138.8 (41.6)	2 (2)
Sipe and colleagues^31^ (2002)	218 (94)					0 (0)
		Midazolam	4.7 (1.5)	Meperidine	89 700 (29 100)	3 (8)
Steenholdt and colleagues^32^ (2020)	342 (139)					4 (6)
		Midazolam	2.4 (0.5)	Fentanyl	60 (20)	3 (5)
Toklu and colleagues^33^ (2009)	159.8			Remifentanil	170	16 (53)
		Etomidate	20.2	Remifentanil	223.2	2 (7)
Ulmer and colleagues^34^ (2003)	277 (105)					4 (8)
		Midazolam	7.2 (2.6)	Fentanyl	117 (30)	4 (8)

Confidence intervals were calculated using the Mantel–Haenszel fixed-effect method of meta-analysis.

**Fig 3:**
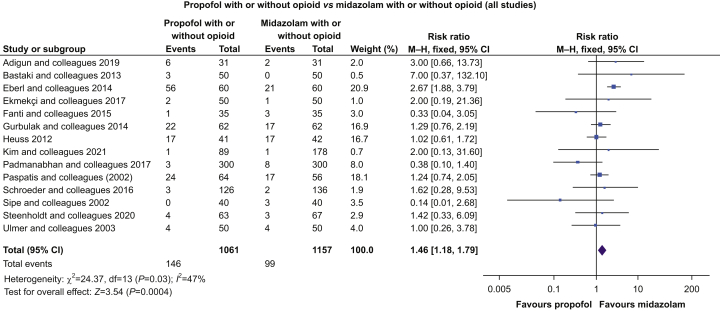
Propofol sedation *vs* midazolam. Risk ratios and 95% confidence intervals (CIs) were calculated using the Mantel–Haenszel (M–H) fixed-effect method of meta-analysis. Hypotension was significantly more frequent with propofol.

Our RoB 2 evaluation (Supplementary material) identified that incomplete blinding and lack of clarity about any analysis plan were common. All 18 studies were included in our analysis. The meta-analysis identified an increased RR for the development of hypotension in procedures where propofol was used compared with the use of etomidate (two studies; *n*=260; RR 2.0 [1.37–2.92]; *P*=0.0003), remimazolam (one study; *n*=384; RR 2.15 [1.61–2.87]; *P*=0.0001), midazolam (14 studies; *n*=2218; RR 1.46 [1.18–1.79]; *P*=0.0004) (Fig. 3), or all benzodiazepines (15 studies; *n*=2602; 1.67 [1.41–1.98]; *P*<0.00001). Hypotension was less likely with propofol than when dexmedetomidine was used (one study; *n*=60; RR 0.24 [0.009–0.62]; *P*=0.003).

## Discussion

We used existing data and publications to scope a potentially important complication of procedural sedation with propofol.

### Prevalence and magnitude of hypotension

By pooling individual patient data from five trials and a clinical database, we have established that hypotension is common with this technique (35% of 939 procedures). Longer periods of propofol sedation and larger propofol doses were associated with longer-lasting and more profound hypotension.

### Comparison with intraoperative hypotension

In a 2007 database study of 15 509 adults having anaesthesia for noncardiac surgery, 49% had one or more episodes of systolic BP <90 mm Hg for 5 min and 31% for 10 min.^36^ More recently, of 23 140 patients undergoing noncardiac surgery at the Cleveland Clinic, whose BP was recorded invasively, only 4791 (21%) had no systolic BP measurements below 90 mm Hg, and the authors identified 5 min <90 mm Hg as the threshold for associated myocardial and renal injury.^3^ We may therefore reasonably conclude that patients having colonoscopy with propofol procedural sedation are exposed to periods of hypotension of a magnitude and duration associated with harm in surgical patients.^4^

A Perioperative Quality Initiative (POQI) consensus statement^37^ concluded that ‘…even brief durations of systolic arterial pressure <100 mm Hg and MAP <60–70 mm Hg are harmful during non-cardiac surgery’. Mean and systolic pressures appear to be equally associated with cardiac and renal injury.^3^ At present, these relationships should be taken to apply only to surgical patients. Importantly, all the reports identify association rather than causation (i.e. the hypotension might reflect comorbidity, which independently precipitates poor outcomes). One signal that hypotension is directly harmful, at least in high-risk patients, was the demonstration by Futier and colleagues^38^ that aggressive control of BP decreased organ injury with the implication that hypotension and at least 25% of major complications are causally related. However, although data evidencing benefit for chasing intraoperative BP targets are sparse, there are none at all for treating hypotension during procedural sedation.

### Comparing propofol and other hypnotics

Having established that propofol sedation for colonoscopy is associated with hypotension, we used focused meta-analysis to confirm that such hypotensive episodes occur frequently and to explore how propofol compares with other hypnotics. Eighteen reports of RCTs comparing propofol with other hypnotics included a total of 1411 patients sedated with propofol of whom 305 (22%) developed one or more episodes of hypotension according to author-defined criteria, typically systolic BP <90 mm Hg. These episodes occurred more frequently when propofol was used for sedation rather than etomidate or the benzodiazepines midazolam or remimazolam. Dexmedetomidine was associated with more hypotension than propofol.

### Limitations

Our analysis is confined to elective colonoscopy, which is a subset of procedural sedation. We chose this approach because colonoscopy with propofol sedation has become very common, and strong demand suggests that the volume of this procedure is likely to continue increasing. Patients undergoing colonoscopy are often older patients with comorbidities and may present in a dehydrated state. They are therefore potentially vulnerable to adverse consequences of hypotension.

The individual patient data, which we were able to access, were collected by multiple groups in different centres using differing equipment, time intervals, and definitions. Not all the data we wanted were available, and although some could be inferred (e.g. dose as mg kg^−1^ from average dose and average weight), some could not. Some individual patient data were sourced from studies investigating interventions (fluid administration and lighter sedation) intended to reduce hypotension. If any of these interventions were effective, this would reduce the amount of propofol-related hypotension. Conversely, granular data collection with shorter than routine intervals between consecutive pressure measurements would likely increase the number of hypotensive episodes detected.

In our focused meta-analysis, we could only include trials that reported hypotension as an outcome. Where the focus of the research lay elsewhere (e.g. patient satisfaction), the decision to report adverse cardiorespiratory outcomes may have been influenced by whether or not they had occurred—reporting bias.

Although a unified set of endpoints for trials in perioperative medicine^39^ remains a work in progress, we cannot expect sedation trialists to be consist in how they describe hypotension—and they are not (Table 1). Nevertheless, hypotension is inevitably reported as episodes of some minimum duration for which the (usually) systolic arterial pressure fell below (usually) 90 mm Hg or (usually) 20–25% below ‘baseline’. Thus, a patient with an episode of systolic hypotension of 89 mm Hg for 5 min would be recorded as equivalent (for this purpose) to another whose pressure fell to 80 mm Hg and took 15 min to restore with fluid and several doses of a pressor. As the association of organ injury with intraoperative hypotension is time and magnitude dependent,^40^ presumably the same might apply if sedation-induced hypotension is other than benign.

Patients in the contributing trials received a variety of opioids or in some cases none at all. In addition, a minority received premedication before sedation with propofol. This heterogeneity of protocol design reflects the lack of clinical consensus about optimal drug choices in procedural sedation. In most, but not all cases, these non-propofol elements were common to the randomised hypnotic treatments. Nevertheless, they reflect the ‘real-world’ deployment of procedural sedation with propofol.

Patients who control their own sedation receive smaller doses of propofol than is administered by third-party sedationists.^15^ Including such patients in an analysis of individual patient data contributes to describing any dose–response relationship in the association with hypotension, and such studies were eligible to include in our data pooling.

In contrast, for our meta-analysis, we excluded studies comparing patient-controlled propofol sedation with alternative hypnotics administered by a sedationist because the outcome data would reflect the sedation strategy and the clinical pharmacology of the study drugs.

Few studies were fully blinded (i.e. one or more of patient, seditionist, and proceduralist were aware of the treatment allocation). This is not surprising. Propofol is a white emulsion typically administered with large syringes or a pump, whereas the comparators are clear solutions whose default concentrations support dosing with smaller volumes. Further, the differing pharmacokinetic and pharmacodynamic characteristics of individual agents make it challenging to devise realistic titration schemes. For these reasons, a lesser degree of blinding is common. Typically, the study data are collected independently of the sedationist with the proceduralist and the patient supposedly unaware of the allocated hypnotic. As the measurement and recording of BP are typically automated, collection of these data might be relatively unaffected by vagaries in the arrangements for blinded sedation.

In a meta-analysis using a fixed-effect model, there is an underpinning assumption that the patients in individual trials are from a coherent overall population and the intervention studied has a fixed effect on individuals to whom it is applied. Table 7 suggests that the characteristics of our patient populations were indeed reasonably similar. Similarly, the pharmacology of i.v. hypnotics is well understood and the concept of an underpinning fixed effect with superimposed random variation is biologically plausible.

### Comparison with previous work

In 2013, Wang and colleagues^41^ reported no differences in cardiopulmonary complications between propofol and non-propofol sedation; however, only four studies related to colonoscopy. Zhang and colleagues^42^ evaluated the propensity to hypotension of propofol and non-propofol sedation schemes on the basis of 808 patients without excluding any studies on quality grounds. The odds ratio of hypotension with propofol was 1.3, but the 95% confidence intervals included the possibility of no effect. The inclusion of studies reporting patient-controlled sedation may have contributed to this conclusion.

### Implications and recommendations for future work

We already know that sedation is not benign and deep propofol sedation may be harmful,^43^ so we should consider how that harm might be reduced. However, a propensity to hypotension—or not—should not be the sole criterion for selecting a hypnotic strategy. Sedationists and proceduralists appreciate the advantageous pharmacokinetics (always) and pharmacodynamics (mostly) of propofol. Further considerations include the quality of patient experience during and after sedation and institutional factors (direct and indirect costs, turnover, and staffing). Equally, given the possibility of propofol-induced harm, we need to be clear why we are using it and when doing so to give no more than necessary.

Although we have identified an association between procedural sedation with propofol and hypotension, we have not demonstrated harm. Hypotension during procedural sedation may not have the same implications as during surgery. Patients undergoing surgery have healing wounds, fluid shifts, pain, and inflammation. Systemic responses or compromise by these factors may be considerably less or absent in patients having procedural sedation. Certainly, their equivalence may not be assumed.

Fortunately, our observations lend themselves to testable hypotheses. Operative hypotension is associated with cardiac and renal injury,^40^^,^^46^ and the injured patients go on to suffer long-term consequences.^47^ Establishing whether these injuries are correlated with sedation-induced hypotension is now a priority. The same methodology—large databases and propensity analysis—could be used to look for immediate and downstream correlations between these organ injuries and episodes of hypotension related to procedural sedation. Even so, correlation does not prove causation, and it is hard to establish that hypotension is causal and not a marker of comorbidity. Finally, if such associations are demonstrable, the question of mitigation arises. Does better BP control reduce adverse outcomes or attenuate their severity? Given a choice between two near-normotensive strategies—minimal (perhaps nurse or patient administered) low-dose propofol and higher doses administered by an anaesthesiologist with fluid and vasopressor support as necessary—is there any difference in adverse outcomes?

For intraoperative hypotension, we now have a developing chain of evidence. It is common and associated with harms,^3^^,^^36^ and attenuating the hypotension mitigates at least a proportion of the harms.^2^ The clinical community has started to accept the evidence, and there is pressure that we adapt our perioperative practice to minimise the risks to patients.^48^ The physiological consequences of procedural sedation have received scant attention to date. We need to take them seriously—or at least to do so, until there is good evidence that we need not bother.

## Authors' contributions

Study conception: all authors.

Writing of first draft: all authors.

Approval of final paper: all authors.
