# Supplementary material for: Hypotension during propofol sedation for colonoscopy: a retrospective exploratory analysis and meta-analysis
Source: Br J Anaesth. 2021 Dec 13;128(4):610–22. doi: 10.1016/j.bja.2021.10.044 (PMC9008870; doi:10.1016/j.bja.2021.10.044)
Supplement: Multimedia component 1 [file mmc1.docx]

**Search**

(((((((((Colonoscopy[MeSH Major Topic]) OR (colonoscop*)) AND ((((((((((((((Deep Sedation[MeSH Major Topic]) OR (Propofol[MeSH Major Topic])) OR (Disoprofol)) OR (Diprivan)) OR (Disoprivan)) OR (Fresofol)) OR (Ivofol)) OR (Recofol)) OR (Aquafol)) OR (2,6-Diisopropylphenol)) OR (Propofol)) OR (monitored-anesthesia-care)) OR (monitored-anaesthesia-care)) OR (deep-sedation))) NOT (case reports[Publication Type])) NOT (editorial[Publication Type])) NOT (guideline[Publication Type])) NOT (introductory journal article[Publication Type])) NOT (review[Publication Type]))

**Risk-of-Bias (RoB 2) summary plot.**

**Procedure time and sedation time**

For following tables, the known mean difference between procedure time and sedation time (3.99 min) was added as a raw number to the procedures where only procedure time was known. Thereafter we ran identical regression analyses as in the main manuscript. The differences are marginal and do not change the overall conclusion.

**Table 4 (+4 min)**

|  | Odds ratio | 95% C.I. | | p-value |
| --- | --- | --- | --- | --- |
|  |  | Lower bound | Upper Bound |  |
| Total dose of propofol (mg kg^-1^) | 1,071 | 1,002 | 1,144 | 0,042 |
| Duration of propofol administration (min) | 1.021 | 1.012 | 1.030 | <0.001 |
| Age (yr) | 0.998 | 0.988 | 1.008 | 0,689 |
| Male sex | 0,945 | 0,712 | 1,255 | 0,698 |
| ASA 1 |  |  |  | 0,635 |
| ASA 2 | 1,021 | 0,726 | 1,436 | 0,906 |
| ASA 3 | 1,234 | 0,762 | 2,001 | 0,393 |

**Table 5 (+4 min)**

|  | Coefficients | S.E. | 95% C.I. |  | p-value |
| --- | --- | --- | --- | --- | --- |
|  |  |  | Lower Bound | Upper Bound |  |
| Total dose of propofol (mg kg^-1^) | -0,625 | 0,272 | -1,159 | -0,091 | 0,022 |
| Duration of propofol administration (min) | -0,219 | 0.032 | -0,282 | -0,156 | <0.001 |
| Age (yr) | 0,153 | 0.042 | 0,071 | 0,234 | <0.001 |
| Male sex | 1,157 | 1,197 | -1,194 | 3,508 | 0,334 |
| ASA PS | -0,553 | 1,028 | -2,572 | 1,466 | 0,591 |

**Table 6 (+4 min)**

|  | Coefficients | S.E. | 95% C.I. |  | p-value |
| --- | --- | --- | --- | --- | --- |
|  |  |  | Lower Bound | Upper Bound |  |
| Total dose of propofol (mg kg^-1^) | 0,534 | 0.215 | 0,112 | 0,956 | 0,013 |
| Duration of propofol administration (min) | 0,227 | 0.025 | 0,178 | 0,277 | <0.001 |
| Age (yr) | -0,013 | 0.036 | -0,084 | 0,058 | 0,716 |
| Male sex | 0,438 | 1,125 | -1,775 | 2,65 | 0,698 |
| ASA PS | 0,498 | 0,994 | -1,457 | 2,453 | 0,617 |

For the following tables we generated a dataset with characteristics of the known difference between the procedure time and the sedation time (mean 3.99 min, SD 3.6 min, n=340, normally distributed) and added the results at random (that is, in the order they were generated) to the procedure times of the study where sedation times where not known. The differences are marginal and do not change the overall conclusion

**Table 4 (+4 min with SD)**

|  | Odds ratio | 95% C.I. | | p-value |
| --- | --- | --- | --- | --- |
|  |  | Lower bound | Upper Bound |  |
| Total dose of propofol (mg kg^-1^) | 1,074 | 1.006 | 1,146 | 0,034 |
| Duration of propofol administration (min) | 1.021 | 1.012 | 1.029 | <0.001 |
| Age (year) | 0.998 | 0.988 | 1.008 | 0,717 |
| Male sex | 0,944 | 0,712 | 1,253 | 0,691 |
| ASA 1 |  |  |  | 0,605 |
| ASA 2 | 1,026 | 0,73 | 1,443 | 0,881 |
| ASA 3 | 1,251 | 0,772 | 2,026 | 0,363 |

**Table 5 (+4 min with SD)**

|  | Coefficients | S.E. | 95% C.I. |  | p-value |
| --- | --- | --- | --- | --- | --- |
|  |  |  | Lower Bound | Upper Bound |  |
| Total dose of propofol (mg kg^-1^) | -0,674 | 0,27 | -1,205 | -0,143 | 0,013 |
| Duration of propofol administration (min) | -0,21 | 0.032 | -0,272 | -0,148 | <0.001 |
| Age (yr) | 0,15 | 0.042 | 0,068 | 0,231 | <0.001 |
| Male sex | 1,176 | 1,199 | -1,179 | 3,53 | 0,327 |
| ASA PS | -0,652 | 1,029 | -2,672 | 1,367 | 0,526 |

**Table 6 (+4 min with SD)**

|  | Coefficients | S.E. | 95% C.I. |  | p-value |
| --- | --- | --- | --- | --- | --- |
|  |  |  | Lower Bound | Upper Bound |  |
| Total dose of propofol (mg kg^-1^) | 0,57 | 0,214 | 0,149 | 0,99 | 0,008 |
| Duration of propofol administration (min) | 0,218 | 0.025 | 0,169 | 0,266 | <0.001 |
| Age (yr) | -0,007 | 0.036 | -0,078 | 0,063 | 0,836 |
| Male sex | 0,39 | 1,129 | -1,829 | 2,609 | 0,73 |
| ASA PS | 0,654 | 0,997 | -1.306 | 2,613 | 0,512 |
|  |  |  |  |  |  |

**Exploration of blood pressure changes**

 
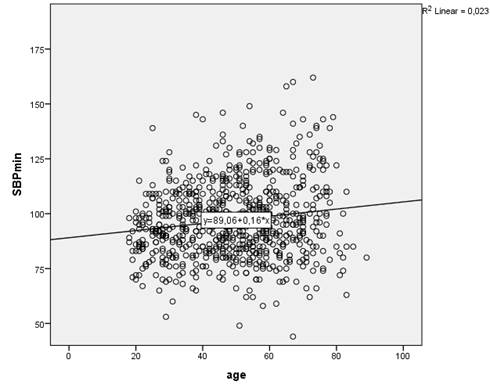


Lowest systolic BP versus age. Lowest SBP is higher in older patients. This is congruent with the regression results (table 5). The coefficient indicates that for every one-year increase in age, the minimum systolic blood pressure increases by 0.16 mmHg

Their drop in SBP, however, was higher in advanced age: delta SBP = difference between baseline (first measurement) and lowest measurement.


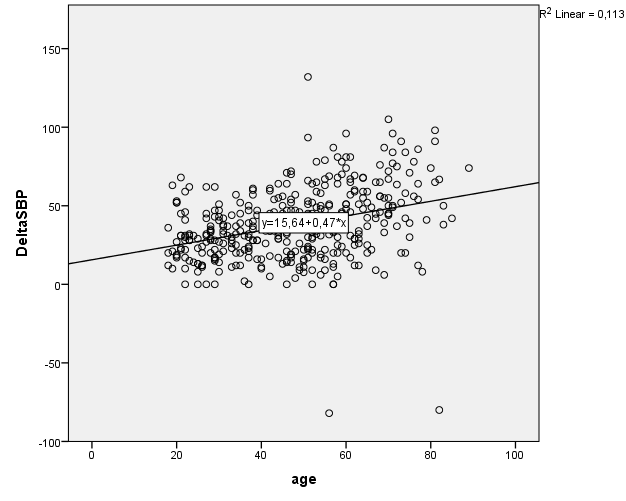


Perhaps because their baseline SBP was higher:


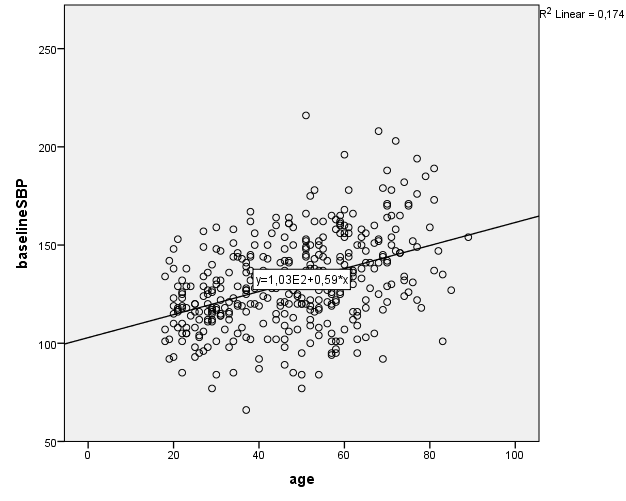


Or perhaps because older people have been given less propofol:


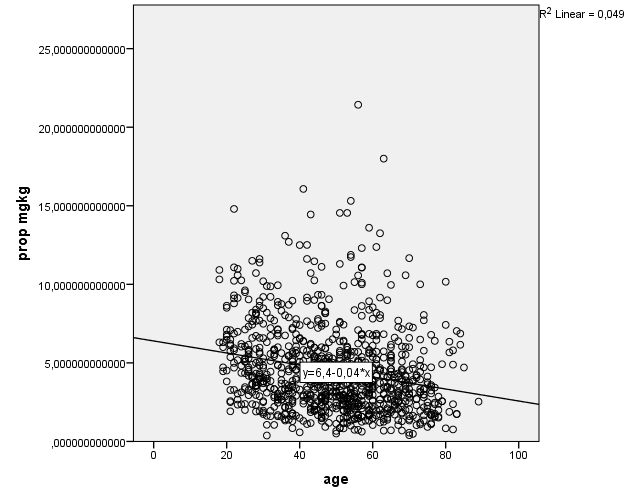


| **Coefficients^a^** | | | | | | |
| --- | --- | --- | --- | --- | --- | --- |
| Model | | Unstandardized Coefficients | | Standardized Coefficients | t | Sig. |
|  |  | B | Std. Error | Beta |  |  |
| 1 | (Constant) | 89.064 | 2.030 |  | 43.872 | .000 |
|  | age | .163 | .039 | .151 | 4.142 | .000 |
| a. Dependent Variable: SBPmin | | | | | | |

 A single regression analysis of age vs SBPmin confirms this.

As does a correlation test:

| **Correlations** | | | |
| --- | --- | --- | --- |
|  | | age | SBPmin |
| age | Pearson Correlation | 1 | .151** |
|  | Sig. (2-tailed) |  | .000 |
|  | N | 939 | 740 |
| SBPmin | Pearson Correlation | .151** | 1 |
|  | Sig. (2-tailed) | .000 |  |
|  | N | 740 | 740 |
| **. Correlation is significant at the 0.01 level (2-tailed). | | | |
